# Regional Blockade of the Shoulder: Approaches and Outcomes

**DOI:** 10.1155/2012/971963

**Published:** 2012-06-25

**Authors:** Clifford Bowens, Ramprasad Sripada

**Affiliations:** Department of Anesthesiology, Vanderbilt University School of Medicine, 1301 Medical Center Drive, 4648 The Vanderbilt Clinic, Nashville, TN 37232-5614, USA

## Abstract

The article reviews the current literature regarding shoulder anesthesia and analgesia. Techniques and outcomes are presented that summarize our present understanding of regional anesthesia for the shoulder. Shoulder procedures producing mild to moderate pain may be managed with a single-injection interscalene block. However, studies support that moderate to severe pain, lasting for several days is best managed with a continuous interscalene block. This may cause increased extremity numbness, but will provide greater analgesia, reduce supplemental opioid consumption, improve sleep quality and patient satisfaction. In comparison to the nerve stimulation technique, ultrasound can reduce the volume of local anesthetic needed to produce an effective interscalene block. However, it has not been shown that ultrasound offers a definitive benefit in preventing major complications. The evidence indicates that the suprascapular and/or axillary nerve blocks are not as effective as an interscalene block. However in patients who are not candidates for the interscalene block, these blocks may provide a useful alternative for short-term pain relief. There is substantial evidence showing that subacromial and intra-articular injections provide little clinical benefit for postoperative analgesia. Given that these injections may be associated with irreversible chondrotoxicity, the injections are not presently recommended.

## 1. Introduction

Common shoulder procedures include hemiarthroplasty, total shoulder arthroplasty, shoulder arthroscopy, subacromial decompression, and shoulder instability procedures such as rotator cuff repair. Anesthesia and analgesia for these surgical procedures are provided by general anesthesia, regional anesthesia, or the combination of general and regional anesthesia. Postoperative pain following shoulder surgery in many patients is severe and may be exacerbated by movement during rehabilitation [1]. Procedures done to help manage the dynamic pain of shoulder surgery include interscalene block, cervical paravertebral block, suprascapular nerve block, subacromial block, and intra-articular injections. Some of these techniques are performed as a single injection, while others are done as a single-injection or continuous infusion. There has been an increase in the number of surgical procedures done in the ambulatory environment. Single-injection and infusion systems utilizing portable, disposable elastomeric pumps help provide safe pain control in this environment. This paper will review the current literature regarding shoulder anesthesia and analgesia. For outcomes, when possible, procedures for shoulder analgesia are compared to the benchmark procedure, the interscalene block.

## 2. Research Method

Medline and PubMed searches were performed for relevant publications regarding shoulder anesthesia and analgesia. Keywords included the following: “shoulder anesthesia,” “shoulder analgesia,” “regional anesthesia,” “interscalene block,” “suprascapular block,” “subacromial block,” and “intra-articular injection.” In addition, the MeSH headings “nerve block” was used. The search was limited to human studies with a concentration on articles occurring in the last five years, but references were included from 1970 to the present. The search identified 259 abstracts that were reviewed. The resulting publication list was then hand-searched, emphasizing randomized controlled trials. One hundred articles were read and reference lists reviewed.

## 3. Shoulder Anatomy and Innervation

The dynamic interaction of bones, joints, muscles, and ligaments establishes the foundation for the unique functions of the human shoulder. The bones which provide the framework for the shoulder girdle are the humerus, scapula, and clavicle. The shoulder girdle consists of three joints and one articulation: sternoclavicular joint, acromioclavicular joint, glenohumeral joint (shoulder joint), and scapulothoracic articulation. The muscles and ligaments of the shoulder allow and restrict movement along with providing active and passive stabilization of the shoulder. Biomechanically, the shoulder has three degrees of freedom and the muscles act at the shoulder to permit particularly free motion: flexion, extension, abduction, adduction, circumduction, internal rotation, and external rotation. Static stability of shoulder is provided by the labrum, capsule, and glenohumeral ligament while dynamic stability is provided by the rotator cuff, long head of the biceps tendon, and periscapular muscles [7].

**Figure 1:**
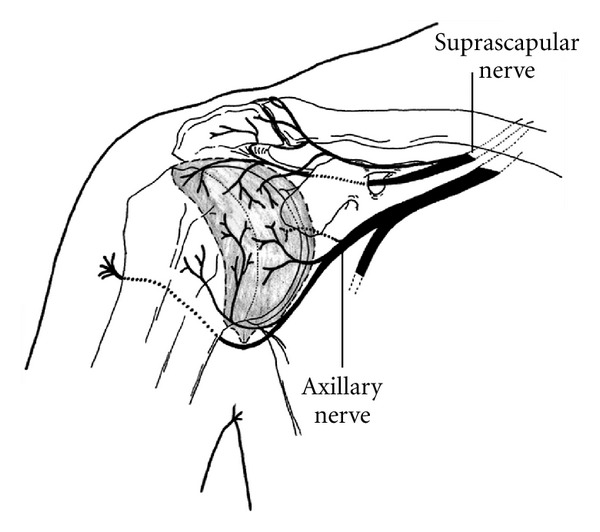
Anterior innervation of the shoulder joint. The suprascapular nerve and axillary nerve are the primary nerves supplying the capsule and the glenohumeral joint (Borgeat and Ekatodramis [1]). (Reprinted with permission from Elsevier.)

**Figure 2:**
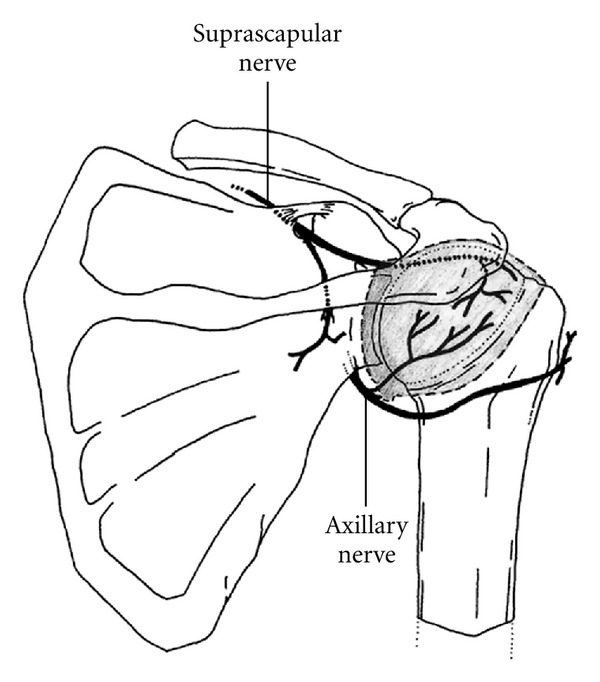
Posterior innervation of the shoulder joint. The suprascapular nerve and axillary nerve are the primary nerves supplying this region (Borgeat and Ekatodramis [1]). (Reprinted with permission from Elsevier.)

The brachial plexus supplies all the motor and most of sensory functions of the shoulder, except the cephalad cutaneous areas of the shoulder, which are innervated by the supraclavicular nerves, originating from the superficial cervical plexus (C3-C4) [1]. Effective control of postoperative shoulder pain generally requires local anesthetic blockade of the nerve supply to the synovium, capsule, articular surfaces, periosteum, ligaments, and muscles of the shoulder [1, 3]. The terminal branches of the brachial plexus that supply the majority of the shoulder innervation are the suprascapular and axillary nerves (Figures 1 and 2).

The suprascapular nerve supplies sensory innervation to the subacromial bursa, acromioclavicular joint, coracoclavicular ligament, and 70% of the shoulder joint capsule [8]. This nerve arises from the superior trunk of the brachial plexus, C5-C6 and possibly C4. The suprascapular nerve descends posteriorly, passing through the scapular notch, innervating the supraspinatus and infraspinatus muscles. It provides sensation for the posterior shoulder capsule, acromioclavicular joint, subacromial bursa, and coracoclavicular ligament.

The axillary nerve originates from C5-C6 nerve roots, with occasional contribution from C4. It is derived from the posterior cord of the brachial plexus. The axillary nerve crosses the anteroinferior aspect of the subscapularis muscle where it crosses posteriorly through the quadrilateral space and divides into two trunks. The anterior trunk supplies the motor innervation to the anterior and middle deltoid muscle. The posterior trunk gives off a branch to the teres minor muscle and the posterior deltoid muscle before terminating as the superior lateral brachial cutaneous nerve, which supplies the cutaneous innervation to the skin overlying the deltoid muscle. 

## 4. Interscalene Block

The interscalene block is the gold standard for shoulder anesthesia and the most commonly used block for shoulder procedures. This approach blocks the brachial plexus at the nerve root or trunk level. Local anesthetic is directed toward C5-C6 nerve roots or the superior trunk. Depending on the volume of local anesthetic used, C7 and even C8 nerve roots may be blocked. The block is especially useful for procedures involving the shoulder, including the lateral two-thirds of the clavicle, proximal humerus, and shoulder joint [1]. Ulnar sparing (C8 and T1 nerve roots) is often seen with this block which limits its usefulness for distal surgical procedures. The interscalene block is performed as a single-injection or continuous peripheral nerve block. The block is done using the paresthesia technique, nerve stimulation technique, ultrasound guidance, or nerve stimulation-ultrasound combination.

### 4.1. Classic Approaches

**Figure 3:**
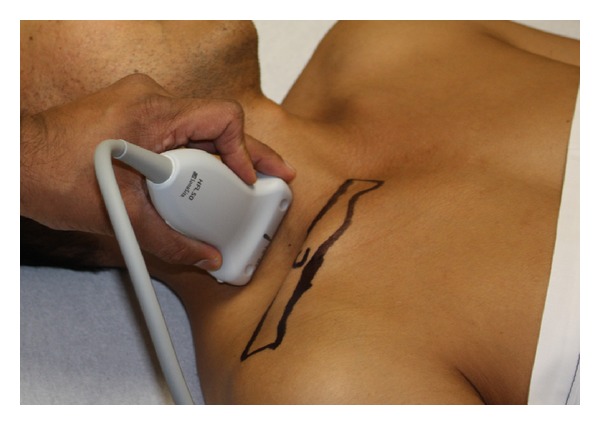
Ultrasound probe position for the interscalene brachial plexus block.

**Figure 4:**
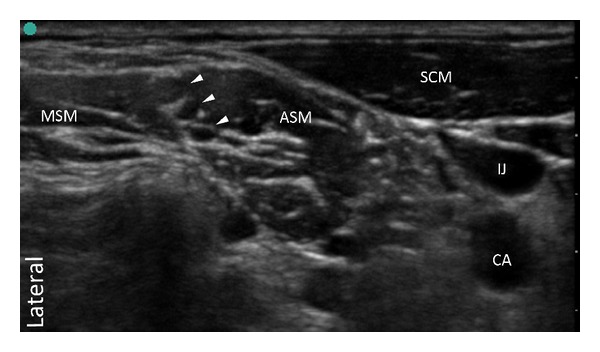
Transverse ultrasound image of interscalene brachial plexus. Arrowheads outline brachial plexus roots within the interscalene groove. SCM: sternocleidomastoid muscle, IJ: internal jugular vein, CA: carotid artery, ASM: anterior scalene muscle, MSM: middle scalene muscle.

The classic approach of Winnie, which is considered the anterior approach, is still commonly performed, especially for single-injection blockade [9]. The technique is performed by palpating the interscalene groove at the level of the cricoid cartilage (C6 vertebra). The needle is directed medially, slightly caudal, and slightly posterior (toward the contralateral elbow) while seeking to elicit a paresthesia in the C5-C6 nerve distribution as the endpoint for injection. The nerve stimulation technique is more commonly used with this approach today, with a relevant motor twitch being the endpoint for injection. Winnie's technique was modified by Meier et al. to reduce complications and to facilitate placement of catheters [10]. Meier used the same landmarks, but enters the skin at 30 degrees and 2-3 cm cephalad to the Winnie approach. The needle is directed toward the middle to lateral third of the clavicle. Borgeat and Ekatodramis also modified the Winnie approach [1]. In this case, the needle is inserted approximately 0.5 cm below the level of the cricoid and directed 45–60 degrees toward the interscalene groove. The Meier and Borgeat modifications, considered lateral approaches to the interscalene block, use the nerve stimulation technique to identify the endpoint for injection. Chan used ultrasound to directly visualize the nerve roots of the brachial plexus in the interscalene groove at the level of cricoid (Figures 3 and 4) [11]. Using ultrasound, the needle is either inserted using an in-plane technique (alignment with the long axis of the probe) or an out-of-plane technique (alignment with the short axis of the probe). While the in-plane technique allows better visualization of the needle, the out-of-plane technique provides a shorter path to target tissues.

### 4.2. Posterior Approaches

**Figure 5:**
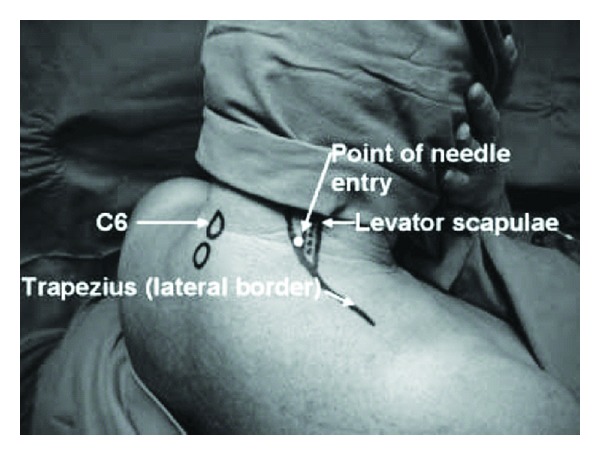
Cervical paravertebral block. Needle entry is at the level of C6, anterolateral to the trapezius muscle and posteromedial to the levator scapulae muscle (Boezaart [2]). (Reprinted with permission from Elsevier.)

The posterior approach to the interscalene blockade has had renewed interest. The cervical paravertebral block was first described by Pippa et al. [12]. Boezaart et al. modified this technique by passing the needle between the levator scapulae and the trapezius muscle to reduce neck pain, which was thought to be due to the needle entering the extensor musculature (Figure 5) [13]. Using the landmarks described by Pippa et al., van Geffen et al. used ultrasound to perform a single-injection interscalene block and Antonakakis et al. used ultrasound to place a catheter for a continuous interscalene block [12, 14, 15]. When using the cervical paravertebral approach, the catheter is better anchored because it passes through multiple layers of muscle and vital anterior structures have less risk of being injured. The major disadvantage of the cervical paravertebral approach is the distance the needle must travel to reach target. The needle must pass through multiple muscle layers which may be painful to the patient. Also, the needle passes through the middle scalene muscle where injury to the long thoracic and dorsal scapular nerves could occur.

### 4.3. Interscalene Block Outcomes

#### 4.3.1. Single-Injection versus Continuous Infusion

Mariano et al. conducted a randomized trial with 30 patients who underwent shoulder surgery that caused moderate-to-severe pain [16]. All patients had a posterior approach, nerve stimulation/ultrasound-guided catheter placement, and a bolus injection of 40 mL of 0.5% ropivacaine. Postoperatively, the patients were discharged with oral analgesics and a portable infusion pump containing either 0.2% ropivacaine or normal saline. The ropivacaine infusion group compared to the normal saline infusion group had greater pain relief, lower supplemental opioid requirements, improved sleep quality, and increased patient satisfaction. Fredrickson et al. performed a randomized study with 68 patients who underwent minor arthroscopic surgery: arthroscopic subacromial decompression, excision of the lateral clavicle, or stabilization [17]. Sixty-one patients included in the study received an ultrasound-guided catheter placement with a bolus of 0.5% ropivacaine. Thirty patients had this catheter removed at the end of the procedure. The other 31 patients received an elastomeric infusion consisting of 0.2% ropivacaine 2 mL/h with a bolus option of 5 mL/h. The continuous infusion group had less pain at rest and with movement during the first 24 hours and lower consumption of Tramadol, but a higher rate of extremity numbness. Kean et al. conducted a clinical trial comparing a single-injection interscalene block to a continuous interscalene infusion [18]. This study only consisted of 16 patients, but revealed that the continuous interscalene infusion group consumed less morphine, had lower visual analogue scores, and overall had higher satisfaction after major surgery of the shoulder. Trompeter et al. assessed 100 patients who underwent shoulder surgery that caused moderate pain [19]. The patients were administered a single-injection interscalene block, followed by general anesthesia for the operation. At discharge, the patients were given a pain diary and followed for 5 days. Fifteen percent of patients experienced severe pain at some point over the first 3 days, but this percentage decreased to 7% by day 5. Ninety-seven percent of the patients were satisfied with their postoperative oral analgesia management and less than 5% contacted their primary care provider for additional analgesia. The authors concluded that a continuous infusion may not be justified for shoulder surgery causing moderate pain.

#### 4.3.2. Neurostimulation versus Ultrasound

Since the arrival of ultrasound as an alternative to performing peripheral nerve blocks, there has been the question, what is better, the nerve stimulation technique or ultrasound? When an accurate image is obtained, ultrasound permits direct visualization of target tissues and surrounding structures. This potentially reduces complications, enhances success rates, and reduces local anesthetic volumes. However, the nerve stimulation technique done for the interscalene block has a high success rate and low complication rate. Furthermore, the nerve stimulation technique is less expensive, more portable and requires less setup time.

Liu et al. performed a randomized trial with 230 patients comparing ultrasound-guided to nerve-stimulation-guided interscalene blocks [20]. Ultrasound reduced the number of needle passes needed to perform the interscalene block and enhanced motor blockade at the 5-minute assessment period; however, there were no differences in block failure, patient satisfaction, or the incidence or severity of postoperative neurological symptoms. Kapral et al. evaluated the success rate of ultrasound compared to nerve stimulation in 160 randomized patients [21]. Surgical anesthesia was achieved in 99% of patients in the ultrasound group compared to 91% of patients in the nerve stimulation group (*P* < 0.01). Furthermore, sensory, motor, and extent of blockade were significantly better in the ultrasound group. Fredrickson et al. conducted a prospective, randomized study in 83 patients comparing ultrasound-guided to nerve-stimulation-guided interscalene catheter placement [22]. The ultrasound group had less needle under the skin time, less need for catheter manipulation after surgery and improved pain scores on the first day, but no difference on the second day.

#### 4.3.3. Local Anesthetic Volumes

One of the advantages of ultrasound-guided nerve blockade is the capacity to place local anesthetic under direct visualization and observe the spread, which allows local anesthetic administration to be adjusted in real time. Since less total volume of local anesthetic may be required to produce an effective block, this could reduce the risk of local anesthetic systemic toxicity [23]. McNaught et al. performed a randomized study with 40 patients comparing ultrasound to nerve stimulation to determine the minimum volume of local anesthetic needed for a successful interscalene block [24]. The minimum effective analgesic volume for the ultrasound group was 0.9 mL compared to 5.4 mL for the nerve stimulation group (*P* = 0.034). Fredrickson et al. conducted a three-staged study that estimated the volume and concentration of interscalene ropivacaine that would prevent recovery room pain after shoulder surgery under general anesthesia [25]. Comparing 20 mL of 0.375% ropivacaine (new, experimental dose) to 30 mL of 0.5% ropivacaine (standard dose), there was no difference in pain or grip strength, but satisfaction was slightly higher with the lower dose. Riazi et al. studied 40 patients randomized to receive an ultrasound-guided single-injection interscalene block using either 5 or 20 mL of 0.5% ropivacaine [26]. The incidence of hemidiaphragmatic paresis was significantly lower in the low-volume group compared to the standard-volume group (45% versus 100%). Postoperative oxygen saturation was higher in the low-volume group (−1.50 versus −5.85, *P* = 0.004). There were no differences in pain scores, sleep quality, and total morphine consumption during the first 24 hours after surgery. Renes et al. studied 30 patients that received a low (C7 level) interscalene block by ultrasound guidance or nerve stimulation [27]. In each group, 10 mL of 0.75% ropivacaine was administered. The incidence of hemidiaphragmatic paresis was lower in the ultrasound group (13% versus 93%, *P* < 0.0001). However, Sinha et al. randomized 30 patients to receive either 20 mL or 10 mL of 0.5% ropivacaine for an ultrasound-guided interscalene block performed at the level of the cricoid cartilage (C5-C6 roots) [28]. The reduction in local anesthetic volume did not reduce the incidence of hemidiaphragmatic paresis, which occurred in 93% of patients in each group.

### 4.4. Complications

Common complications of the interscalene nerve block include phrenic nerve blockade (hemidiaphragmatic paresis), Horner's syndrome, recurrent laryngeal nerve blockade, and vasculature puncture (hematoma). Rarer, but potentially devastating, complications include carotid artery puncture and intervertebral artery injection, pneumothorax, subdural injection, intervertebral foramina injection resulting in spinal or epidural anesthesia, and nerve injury. In addition, indwelling catheters may become infected, kinked, knotted, or entrapped [29, 30]. The risk for most of these complications is minimized by awareness of the location of the needle and surrounding structures. This is facilitated with ultrasound guidance, but even with landmark techniques, the clinician should be aware of areas that are at high risk.

Interscalene blocks and perineural catheters have a risk of complications, but most of these are minor and resolve without sequelae [31–36]. However, the possibility of catastrophic complications exists especially since the interscalene brachial plexus block brings the needle so close to neuraxial structures. Voermans et al. reported a case of permanent loss of cervical spinal cord function associated with the posterior approach to the interscalene block [37]. This was thought to be due to a direct intrathecal and intramedullary injection. Aramideh et al. reported a case in which a patient remained conscious, but developed total paralysis and Horner's syndrome after a brachial plexus block utilizing a posterior approach [38]. Finally, Benumof reported four cases in which the performance of interscalene block during general anesthesia was followed by total spinal anesthesia and extensive permanent loss of bilateral cervical spinal cord function [39].

### 4.5. Summary

Shoulder procedures producing mild-to-moderate pain may be managed with a single-injection interscalene block. However, studies support that moderate-to-severe pain, lasting for several days, is best managed with a continuous interscalene block. This may cause increased extremity numbness, but will provide greater analgesia, reduce supplemental opioid consumption, and improve sleep quality and patient satisfaction. Nerve stimulation is still a good technique for performing an interscalene block. Ultrasound may improve the efficiency of performing blocks since studies reveal less need to manipulate the needle. Also, some studies have shown an improved success rate and faster interscalene block onset with ultrasound. Ultrasound can reduce the volume of local anesthetic needed to produce an effective interscalene block. Blockade performed at the C6 level did not lead to a decrease in hemidiaphragmatic paresis. However, a block performed at the C7 level and volumes less than 10 mL did show a substantial decrease in hemidiaphragmatic paresis, but did not eliminate this problem. Major complications with the interscalene block are rare, but the capacity to view the needle with ultrasound may be beneficial in reducing complications. Presently, it has not been shown that ultrasound offers a definitive benefit in preventing major complications when compared to the nerve stimulation technique.

## 5. Suprascapular Nerve Block

The suprascapular nerve block combined with an axillary nerve block may provide an efficacious alternative to the interscalene nerve block for shoulder anesthesia. The axillary nerve block as described in this section refers to blockade of a terminal branch of the brachial plexus, the axillary nerve. As previously mentioned, the majority of the nerve supply to the shoulder is provided by the suprascapular and axillary nerves (Figures 1 and 2). When these nerves are blocked separately, there may be fewer complications and side effects than the traditional interscalene block [3, 4]. The phrenic nerve is not blocked; therefore, these blocks may be used for patients that are not candidates for an interscalene block, for example, severe chronic obstructive pulmonary disease or contra-lateral hemidiaphragmatic paresis. This technique may also be used as a rescue block for unsuccessful interscalene blocks. A disadvantage of these blocks is that branches proximal to the injection site or regions not innervated by these nerves are not blocked. This may leave analgesia to the surgical region incomplete, requiring supplementation by intravenous analgesics, local anesthetic infiltration, or general anesthesia.

### 5.1. Approaches

**Figure 6:**
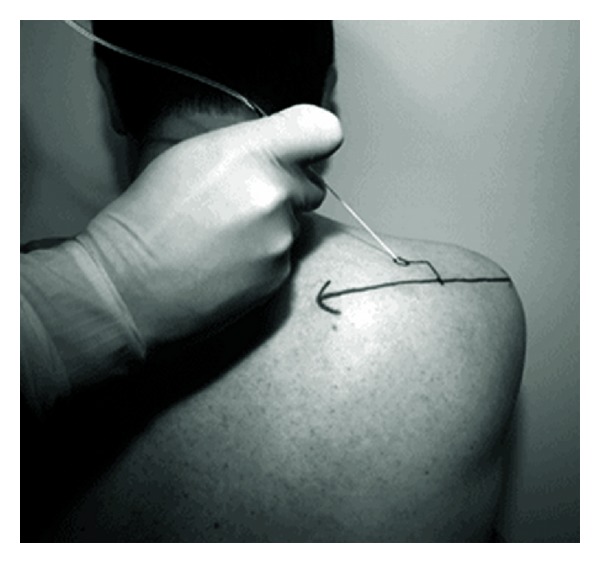
Meier technique for the suprascapular nerve block. Needle insertion is 2 cm cephalad and 2 cm medial to the midpoint of a line connecting the lateral acromion and medial border of the spine of the scapula. The needle is angled 45° in the coronal plane, with 30° of ventral inclination (Price [3]). (Reprinted with permission.)

**Figure 7:**
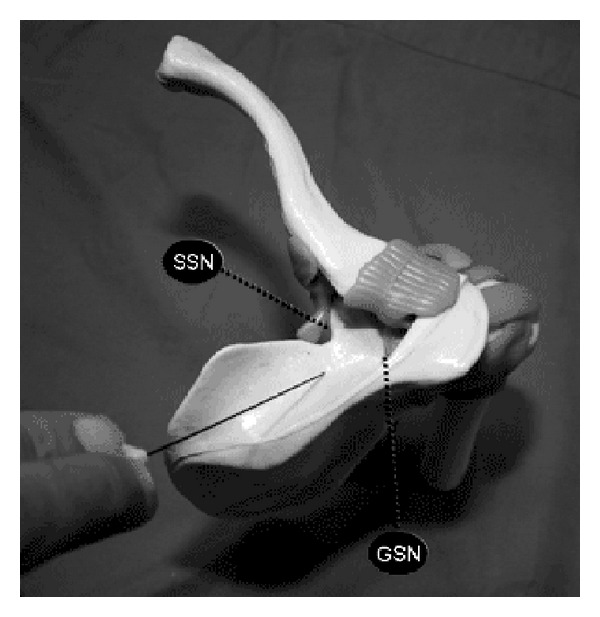
Anatomic representation of the superior view of the Meier technique. Note the needle is in the supraspinous fossa, demonstrating the 30° of ventral inclination. The suprascapular nerve enters the groove at the suprascapular notch (SSN) and winds laterally around the greater scapular notch (GSN) (Price [3]). (Reprinted with permission.)

**Figure 8:**
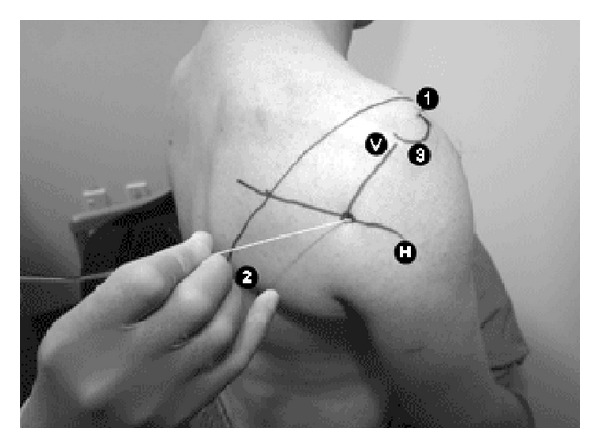
Price technique for the axillary nerve block. A line connects the anterior acromion (1) with the inferior angle of the scapula (2). The midpoint of the line represents the level of the horizontal axis (H) of the quadrilateral space. The line representing the vertical axis (V) is drawn down from the posterolateral aspect of the acromion (3) (Price [3]). (Reprinted with permission.)

**Figure 9:**
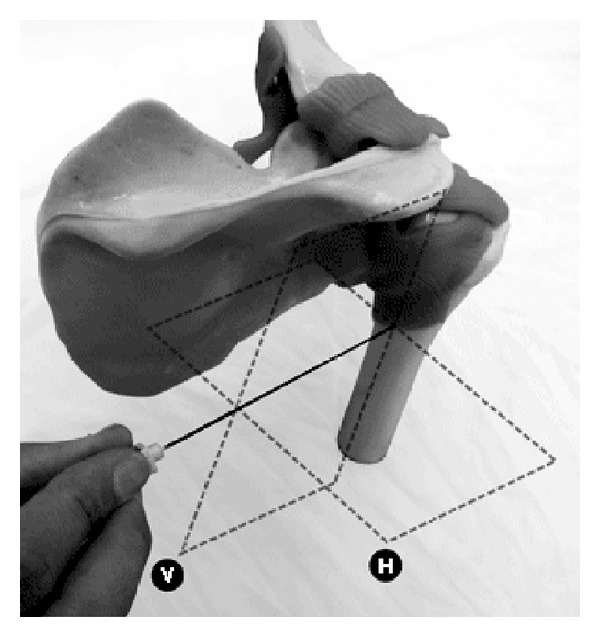
Anatomic representation of the Price technique. The horizontal axis (H) lies at the level of the quadrilateral space, where the axillary nerve passes beneath the glenohumeral joint capsule. The interception of the vertical axis (V) with the horizontal axis (H) allows location of the axillary nerve as it crossing the posterior neck of the humerus (Price [3]). (Reprinted with permission.)

**Figure 10:**
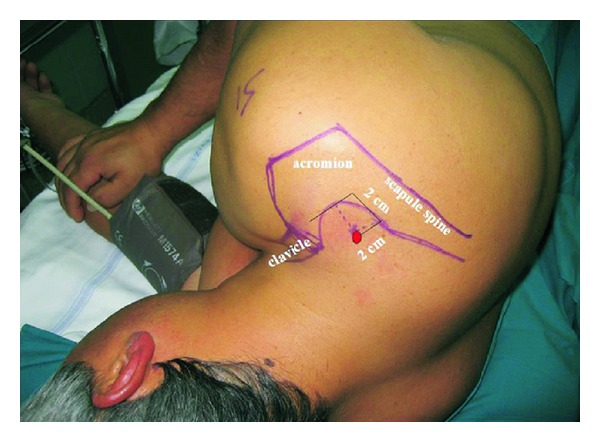
Checcucci technique for the suprascapular nerve block. Needle insertion is 2 cm medial to the medial border of the acromion and 2 cm cephalad to the superior margin of the scapular spine (Checcucci et al. [4]). (Reprinted with permission from Elsevier.)

**Figure 11:**
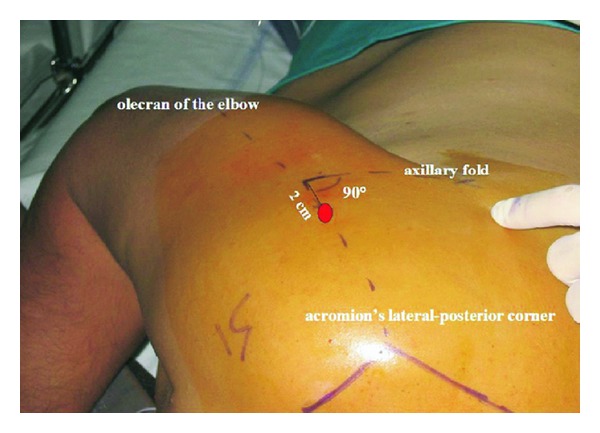
Checcucci technique for the axillary nerve block. A line is drawn between the posterolateral angle of the acromion and the olecranon tip of the elbow. The needle insertion is 2 cm cephalad to the convergence of this line with the perpendicular line originating from the axillary fold (Checcucci et al. [4]). (Reprinted with permission from Elsevier.)

**Figure 12:**
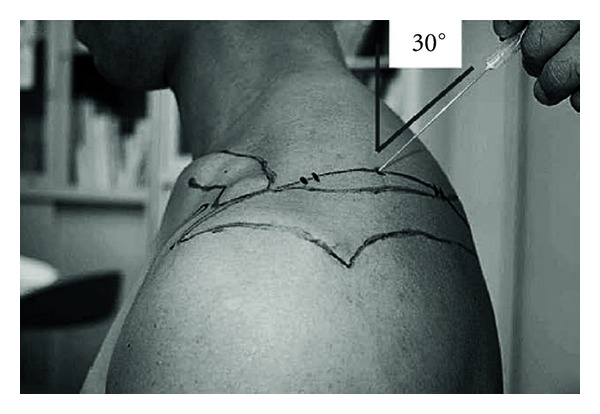
Matsumoto technique for the suprascapular nerve block. Needle insertion point is the midpoint of a line connecting the anterolateral edge of the acromion and the superomedial angle of the scapula. The needle is advanced, at an angle 30° dorsal to the coronal plane, to make contact with the base of coracoid process (Matsumoto et al. [5]). (Reprinted with permission from Elsevier.)

**Figure 13:**
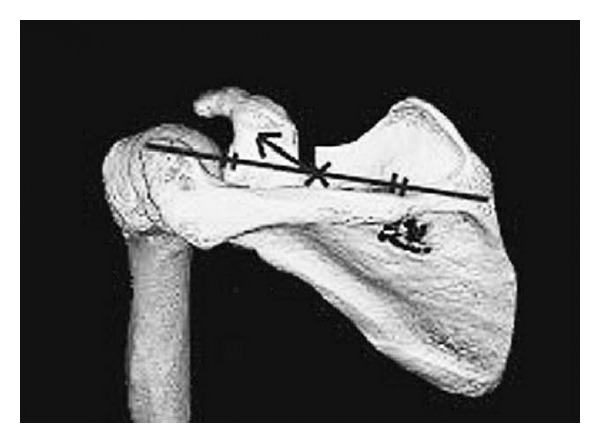
Anatomic representation of the Matsumoto technique (Matsumoto et al. [5]). (Reprinted with permission from Elsevier.)

**Figure 14:**
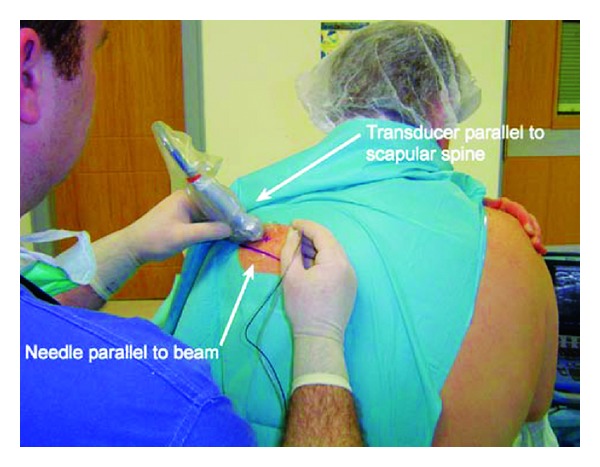
Ultrasound probe and needle position for suprascapular nerve block (Harmon and Hearty [6]). (Reprinted with permission.)

**Figure 15:**
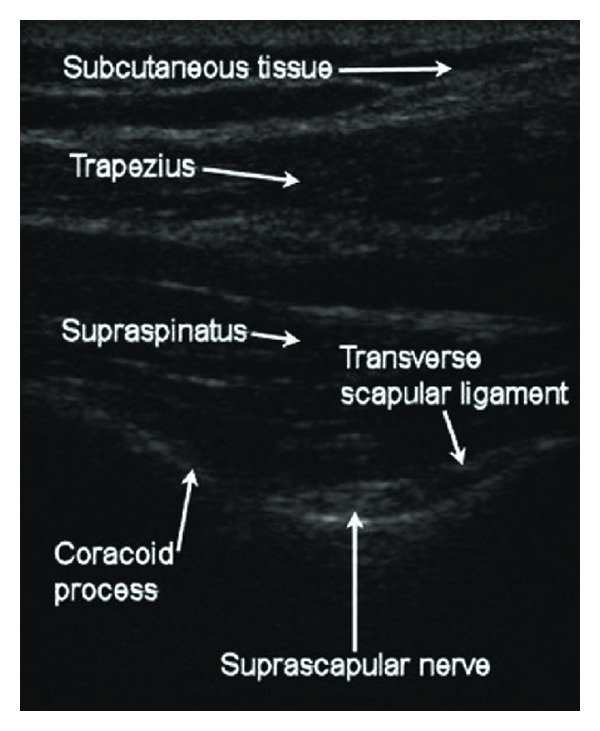
Transverse view of suprascapular fossa and suprascapular notch (Harmon and Hearty [6]). (Reprinted with permission.)

For the suprascapular nerve block, the ideal approach ensures blockade of the more proximal branches to the acromion and the subacromial region to maximize coverage. This may be achieved by blocking the nerve in the suprascapular notch; however, this location is associated with a small risk of pneumothorax [3, 40]. Price described a technique for this block that was adopted from Meier et al. in which the suprascapular nerve is blocked as it travels across the supraspinous fossa (Figures 6 and 7) [3, 41]. In addition, Price described a technique to block the axillary nerve immediately after it passes through the quadrilateral space as it lies just posterior to the humerus (Figures 8 and 9) [3]. Checcucci et al. also described techniques for blocking the suprascapular and axillary nerves [4]. The suprascapular nerve is blocked by eliciting a supraspinatus and infraspinatus motor response (arm abduction and external rotation) after inserting a nerve-stimulating needle at a point 2 cm medial to the medial border of the acromion and approximately 2 cm cephalad to the superior margin of the scapular spine (Figure 10). The axillary nerve is blocked by drawing a line between the lateral-posterior angle of the acromion and the olecranon tip of the elbow. A perpendicular line is drawn from this line to the axillary fold. A nerve-stimulating needle is inserted 2 cm cephalad to the intersection of the lines and a deltoid muscle motor response is sought to identify the axillary nerve (Figure 11). Matsumoto et al. developed a technique for performing the suprascapular nerve block based upon cadaveric anatomy (Figures 12 and 13) [5]. The insertion point is the midpoint of the anterolateral angle of the acromion and the medial edge of the scapular spine. The needle is inclined at a 30-degree angle dorsal to the coronal plane (axis of the body) and inserted until it reaches the base of the coracoid process. Finally, Harmon and Hearty described an ultrasound technique for blocking the suprascapular nerve (Figures 14 and 15) [6]. The ultrasound probe, oriented transversely to the scapula spine, is moved cephalad and lateral to directly visualized and inject around the nerve in the suprascapular notch.

### 5.2. Suprascapular Nerve Block Outcomes

Singelyn et al. conducted a randomized trial of 120 patients comparing an interscalene nerve block to a suprascapular nerve block, intra-articular injection, and control group [42]. There was no difference between the intra-articular injection group and the control group. The suprascapular and interscalene block groups had lower pain scores compared to the intra-articular injection and control groups. The interscalene group had better pain relief with movement than suprascapular group. Overall, the interscalene group had decreased opioid consumption and better patient satisfaction. Jerosch et al. performed a randomized trial of 260 patients who had shoulder operations which caused moderate-to-severe pain [43]. The patients either had a suprascapular nerve block or no block (control). The results showed that from day 1 to day 3 the suprascapular group had significantly better pain scores; however, pain score differences between the groups were relatively small. Jeske et al. conducted a randomized study of 45 patients who had arthroscopic subacromial decompressions performed [44]. The patients were placed in 3 groups: placebo (suprascapular nerve block with 10 mL of 0.9% saline), suprascapular nerve block with 10 mL of 1% ropivacaine, or subacromial infiltration with 20 mL of 1% ropivacaine. The suprascapular group had significantly lower pain scores at 6 h and 24 h, better range of motion, and patient satisfaction. Reported pain scores in the subacromial infiltration group were worse than the placebo group.

### 5.3. Complications

The suprascapular or the axillary nerve blocks have the basic risks of any peripheral nerve block: nerve injury, intravascular injection, and vascular puncture. In addition, the suprascapular block may have a small risk of a pneumothorax.

### 5.4. Summary

The evidence indicates that the suprascapular and/or axillary nerve blocks are not as effective as an interscalene block. Also, these blocks are performed as single injections and have limited use for prolonged postoperative analgesia. However in patients who are not candidates for the interscalene block, these blocks may provide a useful alternative for short-term pain relief.

## 6. Supraclavicular Block

Supraclavicular block provides anesthesia of the entire upper extremity in the most consistent, time-efficient manner of any brachial plexus technique [45]. The “divisions” of the brachial plexus are blocked. Similar to the interscalene nerve block, the block is done between the anterior and middle scalene muscles. The popularity of this approach was limited in the past due to the risk of pneumothorax when using landmark-based techniques [46, 47]. However, ultrasound has rejuvenated interest in this block by providing real-time visualization of the target tissues and surrounding structures, potential reducing complications such as pneumothorax and nerve injury [48, 49]. The block is frequently used for elbow, forearm, wrist, and hand surgery. Since it is performed above the clavicle, it can also provide shoulder analgesia. The concern with using this block for shoulder surgery is that proximal nerves and nerve branches that supply the shoulder may be missed.

### 6.1. Approaches

**Figure 16:**
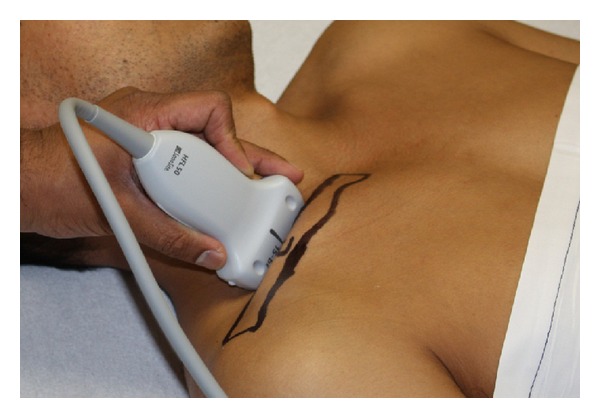
Ultrasound probe position for the supraclavicular brachial plexus block.

**Figure 17:**
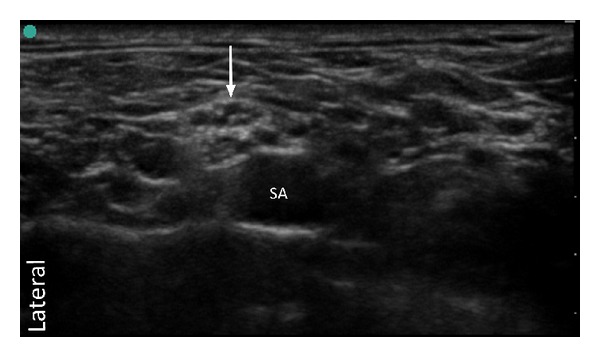
Transverse ultrasound image of supraclavicular brachial plexus. Arrow points toward the divisions of the brachial plexus. The location of the brachial plexus is posterolateral to the subclavian artery (SA).

The supraclavicular block can be performed by two landmark-based techniques, classic approach and plumb-bob approach [45]. Using the classic approach, the needle entry point is 1 cm superior to the clavicle, at the midpoint of the clavicle. It is advanced approximately parallel to the patient's neck and head, from cephalad to caudad toward the first rib. The plumb-bob technique uses an anterior-to-posterior trajectory of the needle to reduce the risk for pneumothorax. With either method, the paresthesia technique or nerve stimulation technique may be used to determine the endpoint for injection. When utilizing ultrasound guidance, a linear probe is placed above and parallel to the clavicle to produce a transverse image of the brachial plexus as it passes just posterolateral to the subclavian artery (Figures 16 and 17) [49]. An in-plane technique (long axis) permits visualization of the entire needle, which allows the provider to direct the needle toward targeted tissues.

### 6.2. Supraclavicular Block Outcomes

Liu et al. conducted a prospective clinical registry of 1,169 patients who underwent shoulder arthroscopy with either an interscalene (*n* = 515) or supraclavicular block (*n* = 654) performed at the discretion of the clinical team [50]. Success rate was excellent for both blocks: interscalene (100%) and supraclavicular (99.7%). The incidence of hoarseness in the postanesthesia care unit was significantly less for the supraclavicular group (22%) compared to interscalene group (31%). The incidence of dyspnea was similar: interscalene (10%) and supraclavicular (7%). There was no clinical evidence of a pneumothorax, and the incidence of postoperative neurological symptoms was low, 0.4%. The authors concluded that ultrasound-guided interscalene and supraclavicular blocks are effective and safe for shoulder arthroscopy.

### 6.3. Complications

Complications of supraclavicular block include pneumothorax (0.6%–6.1%), vascular puncture, intravascular injection, Horner's syndrome, recurrent laryngeal nerve blockade, nerve injury, and phrenic nerve blockade with transient hemidiaphragmatic paresis [51–55].

### 6.4. Summary

There is not enough evidence to support the use of the supraclavicular block for shoulder surgery. The supraclavicular block, similar to the low interscalene block, has a lower incidence of hemidiaphragmatic paresis. Further clinical trials may show the usefulness of the supraclavicular or low interscalene block as an effective alternative to the traditional interscalene block.

## 7. Subacromial Block/Intra-Articular Injections

The subacromial block is generally used for arthroscopic shoulder procedures including subacromial decompression and rotator cuff repair [56, 57]. The pain for these procedures can vary from mild to severe. Therefore, single-injection or continuous infusions are employed depending on the anticipated intensity of the pain. Single-injection and continuous infusions of local anesthetic in the subacromial space have been shown to provide superior pain relief compared to placebo [58–60]. The advantages of subacromial injection (infusion) compared to an interscalene block (infusion) include easy and rapid catheter placement, direct visualization to facilitate proper placement, and little to no extremity numbness or weakness.

Intra-articular injections have many of the same advantages as a subacromial block. However, the efficacy of these injections for postoperative pain management has recently been questioned [42].

### 7.1. Approaches

A subacromial continuous infusion catheter is placed intraoperatively by the surgeon [56]. At the end of the arthroscopy, the posterior portal is used to visualize the subacromial space. Under direct visualization, the catheter sheath is inserted posterolaterally, and several centimeters of catheter are placed in the subacromial space. An elastomeric pump is connected to the catheter for outpatient use. The patient discontinues the pump at home by removing the dressing and catheter. Single injections are done in the same manner without placing the catheter. In the case of intra-articular injections, the glenohumeral joint is directly injected with a local anesthetic, opioid, or combination. This is easily done with direct visualization during arthroscopy.

### 7.2. Subacromial/Intra-Articular Injection Outcomes

Delaunay et al. conducted a randomized trial in 30 patients, who had undergone arthroscopic rotator cuff repair. A subacromial continuous infusion was compared to an interscalene continuous infusion [61]. Both groups received a single-injection interscalene block as their primary anesthetic for the surgery. While the interscalene group had an indwelling catheter placed after the single injection, the subacromial group had a subacromial catheter placed at the end of the surgery. Postoperatively, each patient received a 0.2% ropivacaine continuous infusion (5 mL/h) with a patient-controlled bolus option (5 mL/30 min). The patients were followed for 48 hours. The interscalene group had lower pain scores at rest and with passive motion, decreased morphine consumption, and fewer extra ropivacaine boluses over the 48-hour period. Eleven patients in the interscalene group compared to one in the subacromial group complained of numbness and weakness. Patient satisfaction was comparable between the two groups. Nisar et al. performed a randomized controlled trial of 60 patients who underwent arthroscopic subacromial decompression [62]. The groups consisted of a single-injection interscalene block, single-injection subacromial block, or no block (control). Postoperative pain management was with patient-controlled analgesia (PCA) and oral pain medications. The pain scores in the interscalene and subacromial groups were lower than those in the control group in the first 12 hours postoperatively. The control group consumed more morphine (mean, 32.3 mg) compared with the subacromial group (mean, 21.2 mg) and interscalene group (mean, 14.0 mg) (*P* < 0.001). Winkler et al. conducted a randomized clinical trial in 40 patients who underwent arthroscopic acromioplasty [63]. A subacromial continuous infusion was compared to an interscalene continuous infusion with 0.2% ropivacaine (2 mL/h). The interscalene infusion group had significantly reduced pain scores at rest and during exercise and lower incidence of night pain. Finally, Fontana et al. conducted a clinical trial with 120 patients scheduled for arthroscopy and randomized them to one of five groups: intra-articular injection, subacromial block, interscalene block, intra-articular injection/subacromial block, or control group [64]. All patients underwent general anesthesia. Postoperatively, PCA bolus injections of fentanyl, pain scores, and patient satisfaction were compared. Mean bolus injections and pain scores were as follows: control > intra-articular > subacromial > intra-articular/subacromial > interscalene. However, the patient satisfaction between the intra-articular/subacromial and interscalene block groups was comparable. The authors concluded that a combination of an intra-articular and subacromial block may be a good alternative to an interscalene block.

### 7.3. Complications

One of the advantages of the subacromial injection or infusion may be a lower complication rate in comparison to the interscalene nerve block. Also, there is less risk of extremity numbness or weakness. Busfield et al. conducted a prospective, consecutive study with 583 patients evaluating the short-term complication rate of subacromial infusion pumps [56]. There were no cases of infection, internal catheter breakage, pump failure, or hospital admission for pain control. They concluded that subacromial pain pumps used for arthroscopic shoulder procedures are safe in the short term. Recently, there has been concern about chondrolysis or rapid articular cartilage destruction related to intra-articular injections. Local anesthetics have been shown to induce chondrotoxicity in animal studies, especially when bupivacaine is used in high doses [65]. This is a devastating complication in a young patient and difficult to manage. Even though the cases series by Bailie and Ellenbecker concluded that the etiology of chondrolysis was most likely multifactorial, there was a strong recommendation against the use of large doses of intra-articular local anesthetics [66].

### 7.4. Summary

There is substantial evidence showing that subacromial and intra-articular injections provide little clinical benefit for postoperative analgesia, especially for open and/or rotator cuff procedures. Given that these injections may be associated with irreversible chondrotoxicity, the injections are not presently recommended [8].

## 8. Conclusion

The interscalene brachial plexus block remains the mainstay for shoulder surgery. It is especially beneficial for shoulder surgery causing moderate-to-severe pain. For cases in which pain is anticipated to last for several days, a continuous interscalene block is appropriate. In all the outcome studies in this paper, the interscalene block produced superior analgesia to other techniques. For mild pain, other methods may be considered, but it is still unclear how much benefit these techniques offer in comparison to oral analgesics. When an interscalene block is contraindicated, the suprascapular and axillary nerve blocks offer a reasonable alternative. Since these techniques have not gained widespread use, providers may be less familiar with how to perform them. The suprascapular and axillary nerve blocks appear safe and should be added to the anesthesia provider's skill set. The use of ultrasound in the last five years has enhanced regional anesthesia. Ultrasound-guided upper extremity blockade often provides faster block performance, faster block onset, and greater block success compared to the nerve stimulation technique, while providing a similar safety profile [67]. Finally, intra-articular injection of local anesthetic is not recommended because of the association with chondrolysis.
